# Deciphering DNA replication dynamics in eukaryotic cell populations in relation with their averaged chromatin conformations

**DOI:** 10.1038/srep22469

**Published:** 2016-03-03

**Authors:** A. Goldar, A. Arneodo, B. Audit, F. Argoul, A. Rappailles, G. Guilbaud, N. Petryk, M. Kahli, O. Hyrien

**Affiliations:** 1 Ibitec-S, CEA, Gif-sur-Yvette, France; 2 Université de Lyon, F-69000 Lyon, France; 3 Laboratoire de Physique, Ecole Normale Supérieure de Lyon, CNRS UMR5672, F-69007 Lyon, France; 4 Institut de Biologie de l’Ecole Normale Supérieure (IBENS) CNRS UMR8197, Inserm U1024, 75005 Paris, France; 5 Institut Pasteur, 75015 Paris, France; 6 MRC Laboratory of Molecular Biology, Francis Crick Avenue, Cambridge, CB2 0QH, UK; 7 Biotech Research and Innovation Centre (BRIC), University of Copenhagen, Ole Maaløes Vej 5, Copenhagen 2200, Denmark

## Abstract

We propose a non-local model of DNA replication that takes into account the observed uncertainty on the position and time of replication initiation in eukaryote cell populations. By picturing replication initiation as a two-state system and considering all possible transition configurations, and by taking into account the chromatin’s fractal dimension, we derive an analytical expression for the rate of replication initiation. This model predicts with no free parameter the temporal profiles of initiation rate, replication fork density and fraction of replicated DNA, in quantitative agreement with corresponding experimental data from both *S. cerevisiae* and human cells and provides a quantitative estimate of initiation site redundancy. This study shows that, to a large extent, the program that regulates the dynamics of eukaryotic DNA replication is a collective phenomenon that emerges from the stochastic nature of replication origins initiation.

At the heart of genetic transmission, DNA duplication mechanisms are conserved among eukaryotes^1^. The core of the eukaryal replicative helicase, the MCM2-7 complex, is loaded around DNA in the form of an inactive head-to-head double hexamer (dh-MCM2-7) during the first phase (G1) of the proliferative cell cycle. During the following DNA synthetic (S) phase, a complex reaction, involving several replication factors, activates a fraction of dh-MCM2-7 to form a pair of divergent replication forks that unwind and replicate DNA until they meet with convergent forks assembled at adjacent initiation sites^1^,^2^,^3^,^4^. Initiation sites are called replication origins. Inactive dh-MCM2-7 at the start of S phase correspond to potential origins^5^,^6^,^7^,^8^,^9^. These may become activated later in S phase, or may be unloaded (inactivated) by progressing forks. The mechanisms that determine the location of potential and activated origins remain elusive^10^,^11^. While in *S. cerevisiae*, a unicellular eukaryote, origins are defined by a conserved DNA sequence motif 2, in metazoans no conserved sequence pattern is detected. However, in all eukaryotes the number of potential origins is higher than the number of fired ones^5^. The duration of S phase is finite and the DNA replication process must be completed within a reliable time. This constraint led to the assumption that origins firing is under the control of a deterministic program that regulates their rate and the spatio-temporal pattern of firing^12^,^13^. Recent experimental and theoretical works^14^,^15^,^16^,^17^ challenged this view and suggested that a stochastic firing of randomly distributed potential origins could also meet the temporal constraint imposed by the cell cycle as long as the rate of origin firing increases as S phase progresses^5^,^16^.

The majority of available mathematical and numerical models of DNA replication are founded on an analogy with a one-dimensional crystallization and growth process (KJMA model)^18^. This analogy allows to model replication dynamics by analyzing snapshots of the system to infer its evolution; this model describes the system’s changes of states but not its evolution^16^,^17^,^18^,^19^,^20^,^21^,^22^. Due to the atomistic and geometric nature of the KJMA model in its simplest form, the exact position of fired origins must be defined (localized) to describe the replication dynamics of surrounding regions and the effect of the origin firing propagates along the DNA via the emanating replication forks. Furthermore, in its simplest form, the KJMA model assumes the independence of firing among individual origins and in an arbitrary manner a temporal distribution of origin firing. Thus, these models of DNA replication are adequate to describe the replication process locally but cannot explain how it is influenced by the global compact conformation of the genome. In an effort to link the global conformation of the chromatin to the dynamic of replication, Gauthier & Bechhoefer^23^ developed a model that reproduces the temporal profile of the rate of origin firing by assuming (i) a sequence of three- and one-dimensional replication origin search process for a replication initiation trans-acting factor, and (ii) the independence of firing among individual origins. Along the same line, to reproduce the experimental profile of the rate of origin firing, Goldar *et al.*^19^ have assumed that this rate is regulated by the density of replication forks. The predictions of these models rely on the mechanistic ingredients used to describe the temporal changes of the rate of origin firing per time and per length of unreplicated DNA (*I*(*t*)).

In this paper, we explicitly introduce the unlocalized character of origin firing by picturing the firing process as a transition probability between two possible states (fired or not fired) for any potential origin. We describe the kinetics of replication by using a formal analogy between origin firing in a cell population and scattering process in inhomogeneous media^24^. This point of view does not require to know the exact position or distribution of replication origins along the genome and takes into account the effect of the compact conformation of the genome on the rate of origin firing. Our approach is thus complementary to existing ones: the KJMA model describes the state of a system, our non-local modeling describes the process that leads to the observed state. By considering the experimental observations that (i) dh-MCM2-7 act as potential origins (*m*_0_), (ii) a stochastic process governs their firing and (iii) by the end of S phase a finite number of potential origins (*O*_*total*_) have fired, we predict the temporal profile of the population-averaged number of fired origins as S phase progresses. The outcome of the developed model (i) is in good agreement with experimental observations of parameters describing the kinetics of DNA replication, (ii) confirms the adequacy of equilibrium globule picture to describe the budding yeast chromatin conformation and (iii) can be used to discriminate between two possible pictures to describe the chromatin conformation in human cells.

## Results

**Figure 1:**
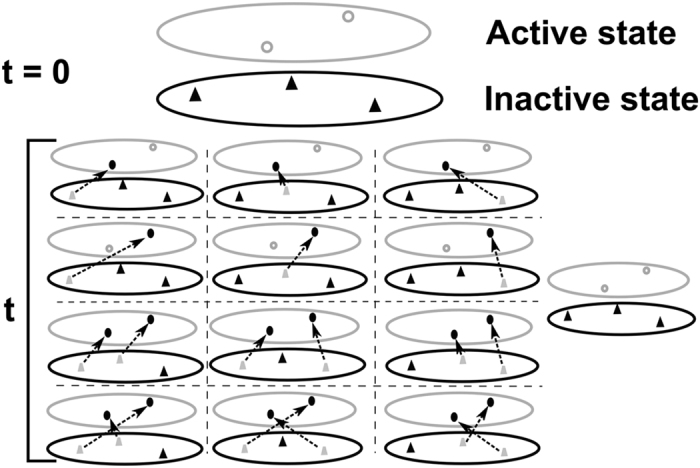
Non localized model of origin firing. *m*_0_ dh-MCM2-7 complexes fill initially (*t* = 0) all positions of inactive state (filled black triangles). The active state is empty (*O*_*total*_ open grey circles). At time *t*, these complexes can transit from inactive state to active state with individual probability rate *ψ* (dashed arrows). By the end of this process, one or several potential origins (filled grey triangles) have fired (filled black circles). However, as potential origins are indistinguishable and the position and the time of fired origins are variable from cell to cell, one cannot designate precisely which potential origin corresponds to which fired origin and one must consider all possible configurations.

As biological observations are performed in a cell population, the genomic positions of potential or fired origins and their firing times are not univocally defined^25^. This leads us to assume that at the start of S phase, there exists a cloud of potential origins (*m*_0_ dh-MCM2-7 loaded on DNA during G1 phase) in each cell. Once the S phase starts, some of them transit from this inactive state to an active state (origin firing) until the end of the S phase where *O*_*total*_ origins are supposed to have fired, leaving *m*_0_ − *O*_*total*_ dh-MCM2-7 in inactive state. We call *k*(*t*) the rate of transition (per potential origin, per time unit and per cell) between the inactive and active state. For simplification, we do not distinguish between the loaded but inactive dh-MCM2-7 state and the unloaded state. Lygeros *et al.*^26^ have previously used the transition probability theory to model replication process in *S. pombe*. By distinguishing loaded but inactive potential origins and unloaded origins, these authors have defined 6 possible states for a potential origin. Here, we model the firing process distinguishing only between fired and non-fired origins. In this 2-state description, the rate of origin firing per cell is equal to the rate of transition, times the number of dh-MCM2-7 that are in inactive state, times the number of free locations in the active state as schematized in Fig. 1:




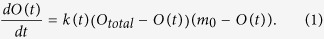




The non local picture of origin firing in a cell population depicted in Fig. 1 implies that each potential origin has the possibility to explore all available configurations in the active state before filling one of them. In other words, two observed fired origins at different times of S phase in a cell population can originate from a common potential origin. Therefore, the calculation of *O*(*t*) is formally similar to the determination of the scattering amplitude in an inhomogeneous media (Supplementary material section 1), where the scattered intensity from two distinct scatterers can originate from a common scatterer^24^. Using this formal analogy and following Matsson’s treatment of the ligand target interaction^27^, the proportion of origin firing per cell at time *t* 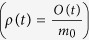 is represented as a Bethe-Salpeter like ladder graph which after summation yields:




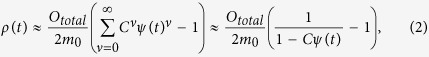




where 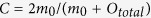, and 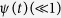 corresponds to the transition probability of an isolated dh-MCM2-7 that is associated in this picture with forward scattering amplitude (see Supplementary material for detailed derivation of Eq. (2)). Note that while *ψ*(*t*) represents the probability of origin firing of an isolated potential origin (low density behavior of the system, meaning no interaction among fired origins), *ρ*(*t*) corresponds to the probability of origin firing considering the cellular context (high density behavior, meaning interaction among fired origins). Direct insertion of Eq. (2) into Eq. (1) together with the change of variable 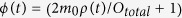 leads to the following compact evolution equation for the observed dynamics of origin firing per cell (Supplementary material section 2):




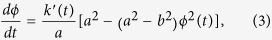




where




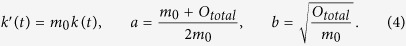




As *ρ*(*t*) is a probability, its values should be always positive, therefore only the forward solution of Eq. (3) has a physical meaning. Using the initial condition that at the start of S phase no origin has fired, we obtain the following general solution:




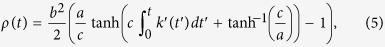




where 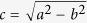.

### Rate of transition *k*(*t*)

*k*(*t*) represents the population-averaged transition rate between the inactive and active states per potential origin. The firing of an origin requires that trans-acting replication factors, that diffuse in the volume defined by the compacted genome (chromatin), find and activate one of the inactive dh-MCM2-7 complexes^25^ that are able to freely diffuse on DNA^28^. Assuming that dh-MCM2-7 complexes are uniformly distributed along the genome, the radius of the volume explored by a dh-MCM2-7 scales as 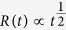. Therefore, the probability *P*_0_(*t*) to find at time *t* a dh-MCM2-7 complex in the nuclear subspace filled by the chromatin is 
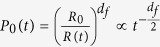
, where *R*_0_ is the characteristic size of the dh-MCM2-7 and *d*_*f*_ is the chromatin’s fractal dimension. The probability to find a trans-acting factor in the fractal structure of chromatin^29^ at time *t* is proportional to 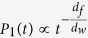, where *d*_*w*_ is the fractal dimension of the trans-acting replication factor’s random walk^30^,^31^. Hence, the probability that in an elementary volume at time *t* a trans-acting factor meets a dh-MCM2-7 is 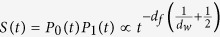. Since the spatial distribution of both dh-MCM2-7 and trans-acting factors are not homogeneous in the volume of the nucleus, the transport process that leads to the encounter between these two actors cannot be neglected. Thus, the rate of transition from inactive to active sites is no longer a time constant *k*_0_ equal to the population averaged probability of origin firing per potential origin and per cell, but it has to be normalized by a fraction of the total number of dh-MCM2-7 and trans-acting factor encounters during the time *t*: 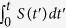. This leads to the following time dependence of the transition rate:




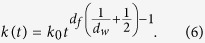




### Fraction of replicated DNA: *f*_*DNA*_(*t*)

To calculate *f*_*DNA*_(*t*), we use the analogy between DNA replication and one-dimensional nucleation and growth phenomena^18^. In this analogy, the firing of a potential origin corresponds to a nucleation event and the propagation of divergent replication forks at constant velocity *v* to a growth event. Following Avrami^32^, we consider the genome at an instant *t*, and assume that *O*(*t*) origins have already fired. The probability that, at time *t*, a particular locus of the genome is not covered by a particular replicon is 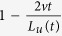, where *L*_*u*_(*t*) is the length of the unreplicated genome. So the probability that it is not covered by any *O*(*t*) replicons is 
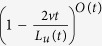
. Assuming that 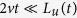, this probability becomes 
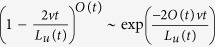
. Finally, the probability that a locus is covered at time *t*, is just the fraction of replicated DNA:




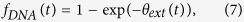




where 
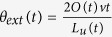
. As firing of origins is an asynchronous phenomenon, in reality 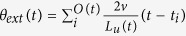, where *i* is an index running over all fired origins. Each origin *i* fires and starts growing at *t*_*i*_. We change the discrete sum on *i* to a continuous integral over time (*t*) and considering that 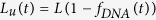, we get for *θ*_*ext*_(*t*):




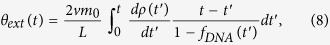




where *L* is the size of the genome.

### Rate of origin firing per unreplicated length of DNA (*I*(*t*)) and fork density (*N*_*f*_ (*t*))

*I*(*t*) is defined as the number of fired origins per unit of time and per unit of length of unreplicated DNA:




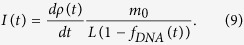




It is interesting to note the similarity between Eq. (9) and the expression of *I*(*t*) derived by Gauthier and Bechhoefer (Eq. (6) in ref. 23). Both expressions of *I*(*t*) are obtained assuming that the trans-acting replication factor diffuses in the volume defined by the chromatin. However while here we consider the collective rate of origin firing 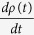, in ref. 23, the authors assume that the origins fire independently (see Supplementary material section 3, last paragraph for more discussion). Following the expression of domain (replication bubble) density calculated by Yang *et al.*^33^, the density of replication forks is obtained under the following integral form:




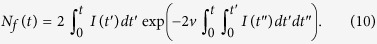




Then by introducing Eqs (5) and (6) into Eqs (7, 8, 9, 10), we show that the dynamics of *f*_*DNA*_(*t*), *I*(*t*) and *N*_*f*_(*t*) during the S phase can be completely characterized by the knowledge of 7 measurable parameters: *m*_0_, *O*_*total*_, *d*_*f*_, *d*_*w*_, *k*_0_, *v* and *L*.

Recent technological developments have facilitated the access to the replication dynamics of *S. cerevisiae* and *H. sapiens* and provide some reliable quantitative estimates of our model parameters. *S. cerevisiae* has a genome of length 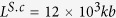 while the size of the haploid human genome is ~280 times larger 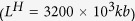. The number of dh-MCM2-7 complexes per cell has been estimated experimentally both in *S. cerevisiae* 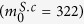
34,35 and human HeLa cells 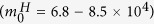
36. In *S. cerevisiae* on average 
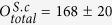
 origins are referenced to fire systematically during a single S phase per cell^37^,^38^. In contrast the number of systematically fired origins in a human cell population is rather poorly known. Recent single-molecule^15^ and genome-wide^3^,^39^ studies estimated that on average between 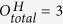 to 9.2 × 10^4^ origins fire per cell cycle. The speed of fork progression was measured experimentally in *S. cerevisiae*^40^ as 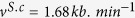 and was deduced from single-molecule and genome-wide replication timing studies of replicating HeLa cells^15^ to range between *v*^*H*^ = 0.8 and 3.5 *kb.min*^−1^. The geometrical fractal dimension *d*_*f*_ and the dynamic fractal dimension *d*_*w*_ can be combined to define the spectral dimension^41^ *d*_*s*_ = 2*d*_*f*_/*d*_*w*_. The spectral dimension characterizes the power-law decay of the intra-chain contact probability of a polymer as 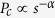, where *s* is the number of monomers along the chain^41^,^42^ and 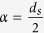. From the experimentally measured distribution of the frequency of intra-chromosomal contact points, one can extract *d*_*s*_. In the case of *S. cerevisiae*, it was experimentally measured that^43^ 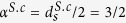. As the conformation of the chromatin inside the yeast nucleus can be reasonably considered to be an equilibrium globule^44^, hence 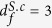 and so 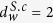 (normal diffusion). In HeLa, the observed the intra-chromosome contact probability was observed to be inversely proportional to the distance between the contact points^45^, 
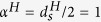
. In HeLa, two different models for chromatin organization inside the nucleus were proposed. The first and historical interpretation is to consider that the chromatin fiber is self-organized into a long-lived, non-equilibrium unknotted conformation allowing easy opening and closing of chromosomal regions over large distances in the nucleus^45^; this interpretation leads to model the chromatin as a “crumple” or fractal globule^44^,^45^. Following this model, as it is independently measured that in HeLa cells^46^,^47^ 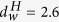 (subdiffusion), we conclude that 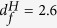 (see Dissussion). The second alternative interpretation is based on the recent analysis of Hi-C data in different human cell types by Boulos *et al.*^48^. By combining an integrative analysis of epigenetic maps and Hi-C data, these authors have shown that the 3D equilibrium globule model with *d*_*f*_ = 3 and *d*_*w*_ = 2 provides a comprehensive description of the Hi-C contact probability power-law exponent 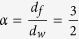 observed in (i) embryonic stem cells as the signature of an accessible and permissive genome structure possibly shaped by pluripotency factors^49^, and (ii) somatic cells between gene rich, early replicating euchromatin pairs of loci confirming that active chromatin in differentiated cell lines is preferentially positioned in the nuclear interior^49^,^50^. Importantly, Boulos *et al.*^48^ have further shown that Hi-C contact probability exponent *α* ≤ 1 is indeed observed in differentiated cells between gene poor, late replicating heterochromatin pairs of loci as an indicator of the confining of this lamina-associated heterochromatin to the nucleus periphery^49^,^50^, consistent with the prediction of the 2D equilibrium globule model *d*_*f*_ = 2, *d*_*w*_ ≥ 2 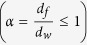. Using this interpretation, we propose that the observed replication signals result from the superposition of the replication dynamics influenced by a 3-D and 2-D equilibrium globule organization of the chromatin fibre. To find the proportion of each signals, we follow the interpretation of Boulos *et al.*^48^ and assume that the signal from 2-D equilibrium globule organization of the chromatin represents only 38% of the total signal, representing the amount of chromatin that interacts with the lamina in a constitutive manner^51^.

Now, using Eq. (5) and the boundary condition that by the end of S phase (*t*_*end*_) 
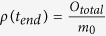
, we obtain




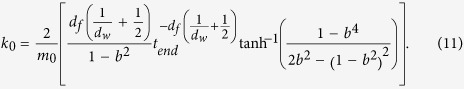




As during S phase, origins are fired in a continuous and irreversible manner^52^, and only once per cell cycle^4^, then 0 ≤ *k*_0_ ≤ +∞ and from Eq. (10), we find the following boundaries to *ρ*(*t*_*end*_):




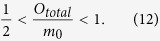




This inequality is verified for *S. cerevisiae* 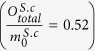, indicating that *ρ*(*t*_*end*_) almost saturates the lower bound in Eq. (12). This observation turns out to be also valid for HeLa cells where the comparison of our model predictions with the replication dynamical data (see below) also selects an origin redundancy 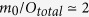 with 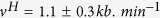, 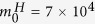 and 
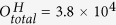
 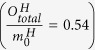. Knowing that 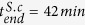
53 and 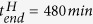
15, we get 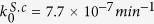 and 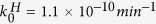.

**Figure 2:**
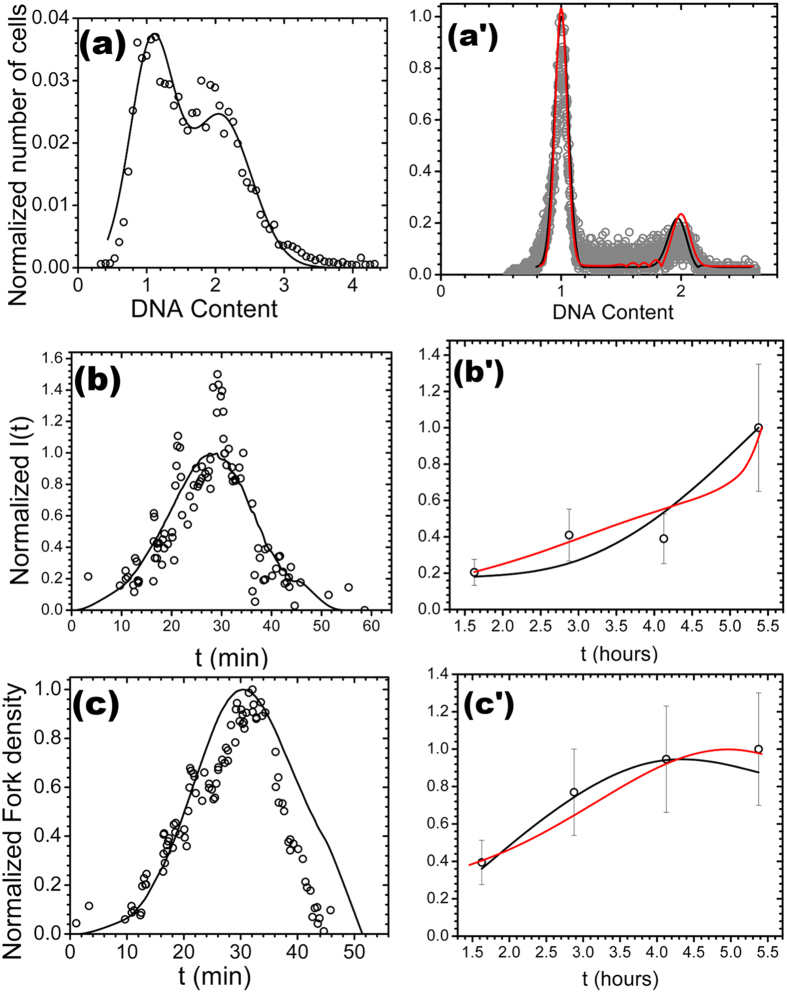
The open circles are experimental data and the solid lines are the calculated profiles. 3D equilibrium globule model of chromatin (black curve; *d*_*f*_ = 3, *d*_*w*_ = 2) for *S. cerevisiae* (data from Ma *et al.*^53^): (**a**) Facs profile calculated from *f*_*DNA*_(*t*)^53^ (*C* = 0.94, *P* < 10^−10^); (**b**) *I*(*t*) (*C* = 0.87, *P* < 10^−10^); (**c**) *N*_*f*_ (*t*) (*C* = 0.89, *P* < 10^−10^). Fractal globule model of chromatin (black curve; *d*_*f*_ = 2.6, *d*_*w*_ = 2.6) for HeLa (data from Guilbaud *et al.*^15^): (**a’**) Facs profile (*C* = 0.94, *P* < 10^−10^); (**b’**) *I*(*t*) (*C* = 0.94, *P* = 5.5 × 10^−12^); (**c’**) *N*_*f*_ (*t*), (*C* = 0.96, *P* = 3.5 × 10^−2^). 3D-2D equilibrium globule organization model of chromatin (red curve; *d*_*f*_ = 3 (62%), 2 (38%), *d*_*w*_ = 2 see text) for HeLa (data from Guilbaud *et al.*^15^): (**a’**) Facs profile (*C* = 0.97, *P* < 10^−10^); (**b’**) *I*(*t*) (*C* = 0.96, *P* = 1.5 × 10^−2^); (**c’**) *N*_*f*_ (*t*), (*C* = 0.93, *P* = 6.3 × 10^−2^). 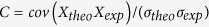 is the Pearson linear correlation coefficient.

Our 7 model parameters being fixed, we use Eqs (5, 6, 7, 8, 9, 10) to numerically calculate *f*_*DNA*_(*t*), the flow cytometry (Facs) profiles, *I*(*t*) and *N*_*f*_ (*t*) for both *S. cerevisiae* and HeLa and compare the obtained theoretical profiles to recent experimental data reported in refs 15  and 53 respectively. As shown in Fig. 2, the agreement between theory and experiment is very good.

## Discussion

The success of this analysis sheds light particularly on two aspects of DNA replication. First, we explicitly link the rate of origin firing to the global conformation of chromatin and to the diffusion of replication factors inside the nucleus. We find that for both considered organisms, the spectral dimension *d*_*s*_ ≥ 2, suggesting that origin firing is only transiently regulated by the random encounter of a transacting factor and a dh-MCM2-7 complex^54^. Furthermore, in both cases the encounter probability *S*(*t*) decreases faster than *t*^−1^, a behavior that is representative of non compact exploration diffusion process (*i.e.* the number of sites explored by the transacting factor is smaller than the number of sites present in the volume defined by the chromatin)^30^. This is not surprising as only the encounter of a transacting factor with an inactive dh-MCM2-7 that is still bounded to a non replicated region of the genome can lead to the transition of the latter to the active state. Second, the irreversibility of replication process involves that the number of fired origins should at least represents half of the potential origins per cell. Note that our model further suggests that if during the S phase less than half of potential origins are used, the rate of transition *k*(*t*) would have a dissipative component (*k*_0_ become a complex number) inducing that by the end of S phase all the genome would not be replicated. The results reported in Fig. 2(b,b’) provide a quantitative estimate of origin redundancy^8^,^9^ in a single cell to 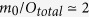. We propose that the finite length of S phase applies an evolutive pressure that fixes 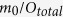.

**Figure 3:**
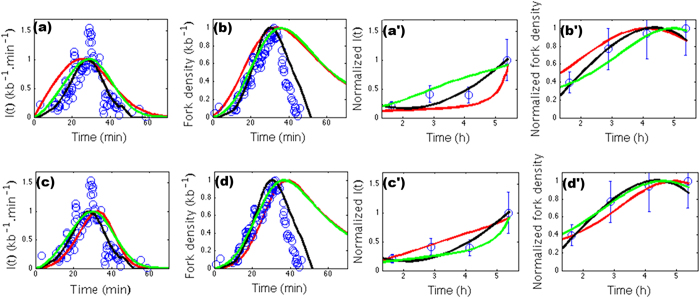
The open circles are experimental data and the solid lines are the calculated profiles. *S. cerevisiae* (data from Ma *et al.*^53^): (**a**) *I*(*t*) and (**b**) *N*_*f*_ (*t*) for different values of the chromatin fractal dimension: *d*_*f*_ = 2 (red), 3 (black) and 2.5 (green). (**c**) *I*(*t*) and (**d**) *N*_*f*_ (*t*) for different values of the dynamics fractal dimension: *d*_*w*_ = 1.5 (red), 2 (black) and 2.5 (green). HeLa (data from Guilbaud *et al.*^15^): (**a**) *I*(*t*) and (**b**) *N*_*f*_ (*t*) for different values of the chromatin fractal dimension: *d*_*f*_ = 2 (red), 2.6 (black) and 3 (green). (**c**) *I*(*t*) and (**d**) *N*_*f*_ (*t*) for different values of the dynamics fractal dimension: *d*_*w*_ = 2 (red), 2.6 (black) and 3 (green).

Profiles of *I*(*t*), *N*_*f*_ (*t*) and *f*_*DNA*_(*t*) are sensitive to the origin usage, but the shape of *I*(*t*) and *N*_*f*_ (*t*) are particularly sensitive to *d*_*f*_ and *d*_*w*_ in both *S. cerevisiae* and Hela (Supplementary material section 4). Importantly, our analysis confirms that the conformation of the chromatin in budding yeast can be represented as an equilibrium globule^44^ in three dimensions (*d*_*f*_ = 3, *d*_*w*_ = 2) (Fig. 3(a–d)), consistent with the observed power-law decay of the intra-chromosome contact probability^43^ with exponent 
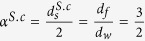
. The scarcity of experimental replication data in Hela cells makes these data less selective for the estimate of 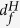 and 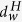 in human (Fig. 3(a’–d’)). This explains that rather equal agreement of the replication data was obtained in Fig. 2(a’–c’) with both the fractal globule model^44^,^45^ and the 3D-2D equilibrium globule model^48^. The consistency in human somatic cells between replication data and the compartmentalization of the genome into an early replicating 3D equilibrium globule euchromatin organization in the nucleus interior and a late replicating 2D equilibrium globule heterochromatin confined at the nuclear envelop requires further investigation of new experimental data.

To conclude, the existing models of DNA replication^16^,^17^,^18^,^21^,^22^ require an *a priori* knowledge of spatio-temporal map of origin firing, and the variability of the latter is treated as a small deviation from their population averaged values. Here, we explicitly consider the variability on the position and firing time distribution of origins in a cell population^14^,^25^ and use a non-local treatment to calculate their rate of firing. This allows us to develop an effective description of DNA replication dynamics using a physical analogy between origin firing and scattering phenomena in an inhomogeneous medium. One of the outcome of such a description is that this dynamics is self-referential. The self-reference arises because we consider the replication process in a cell population, demonstrating that the temporal pattern of DNA replication is emergent and not predefined as in the KJMA theory. Furthermore, the distributed nature of our analysis (Supplementary material, section 3), allows (i) linking the kinetics of observed proportion of fired origins (Eq. (5)) to the averaged rate of single origin firing by taking into account the global topology of the genome inside the cell nucleus through its spectral dimension and (ii) ties the individual probability of firing of an origin to their collective observed probability (Eq. (2)). Therefore this model is a macroscopic model that overpasses the detailed molecular mechanisms necessary to the firing of an origin and retains the minimal necessary steps of DNA replication that are conserved among eukaryotes.

## Additional Information

**How to cite this article**: Goldar, A. *et al.* Deciphering DNA replication dynamics in eukaryotic cell populations in relation with their averaged chromatin conformations. *Sci. Rep.* **6**, 22469; doi: 10.1038/srep22469 (2016).

## Supplementary Material

Supplementary Information
