# Supplementary material for: Deciphering DNA replication dynamics in eukaryotic cell populations in relation with their averaged chromatin conformations
Source: Sci Rep. 2016 Mar 3;6:22469. doi: 10.1038/srep22469 (PMC4776152; doi:10.1038/srep22469)
Supplement: Supplementary Information [file srep22469-s1.pdf]

Supplementary material: Deciphering DNA replication dynamics in eukaryote cell populations in relation with their averaged chromatin conformations

A. Goldar[1], A. Arneodo[2], B. Audit[2], F. Argoul[2], A. Rappailles[3,4], G. Guilbaud[3,5], N. Petryk[3,6], M. Kahli[3] & O. Hyrien[3]

[1]*Ibitec-S, CEA, Gif-sur-Yvette, France*

[2]*Université de Lyon, F-69000 Lyon, France and Laboratoire de Physique, Ecole Normale Supérieure de Lyon, CNRS UMR5672, F-69007 Lyon, France*

[3]*Institut de Biologie de l'Ecole Normale Supérieure (IBENS) CNRS UMR8197, Inserm U1024, 75005 Paris, France*

[4]*Institut Pasteur, 75015 Paris, France*

[5]*MRC Laboratory of Molecular Biology, Francis Crick Avenue, Cambridge, CB2 0QH, U.K.*

[6]*Biotech Research and Innovation Centre (BRIC), University of Copenhagen, Ole Maaløes Vej 5, Copenhagen 2200, Denmark.*

1 Analogy with scattering in inhomogeneous media

Detailed derivation of equations 2. Consider the propagation of a wave through an semi-infinite slab where position and orientation of scattering centers are randomly distributed. The intensity of the incoming beam in a particular direction at a position x of the slab is reduced due to multiple

scattering. Therefore, it is difficult to assign the observed scattered intensity to a particular scattering center. One way to overcome this difficulty is to assume that all existing scattering centers participate in the scattering of the incoming beam, hence the scattering effect of the medium is not anymore *localized* on a single scatterer but is now *delocalized* over the whole medium [1].

In the same manner, consider a cell population. The set of potential replication origins (position and time of firing) are not identical from one cell to another. Therefore, similar to the scattered intensity of the incoming beam, the observation of firing of n replication origins at a time t does not represent the firing of the same potential replication origins in all cells but corresponds to the firing of a subset of replication origins present in the cell population. In that sense, one cannot assign the firing probability to a particular potential origin (*localized*) but one must *delocalize* it over a subset of potential origins.

As an example, let consider a scalar wave (a firing process in the case of replication), we aim at calculating the scattered intensity (observed number of fired origins) at a position x (at an instant t) and by averaging over the whole volume to get the average intensity scattered by the medium (whole genome averaged firing probability). Suppose we have strong scatterers (fired replication origins), in the limit of point scatterers, the scalar wave evolves in a potential $V(r) = -\sum_{i=1}^{N} u\,\delta(x - X_i)$, where $-u$ is the bare scattering strength (intrinsic firing strength) and X_i are the positions of the scatterers randomly distributed in the sample (positions of potential origins). In the bulk of the sample, far from boundaries, one can only detect the intensity of diffuse-scattered beam whose amplitude depends on the realization of disorder (location and orientation of scatterers). It is impossible to calculate in an accurate manner all scattering interactions in the sample.

However, under some approximations, one can reduce the number of interactions to consider, and calculate in a satisfactory manner the intensity of the scattered beam. To define these approximations in relation with replication process, we explicitely take into account the following experimental evidences: i) during replication process an origin cannot fire more than once during a single S phase[2], and ii) the number of fired origins is smaller than the number of potential origins[3]. The first observation can be interpreted as the fact that a scatterer is only visited once (first order Born approximation[4]). The second observation can be considered as the fact that the density of strong scatterers is small and leads to the hypothesis that the intrinsic scattering strength of a scatterer can be replaced by an effective (screened) scattering strength that takes into account all possible scattering paths between scatterers (Ladder approximation[5]). Under these hypotheses, the diffuse intensity at a point x can be written as:

$$I\left(x\right) = n\sigma_{sc}\int d^3x' \left|G\left(x - x'\right)\right|^2 \left|\psi_{in}\left(x'\right)\right|^2, \qquad \text{(Sup.1)}$$

where n is the density of scatters, σ_{sc} is the scattering cross-section, $G\left(x - x'\right)$ is the dressed Green's function and $\psi_{in}\left(x\right)$ is the incoming wave.

The Green's function of the random medium $G\left(x - x'\right)$ (also called the dressed propagator), can be obtained by solving the Dyson equation[6]

$$\left|G\left(x - x'\right)\right|^2 = G_0 + G_0\Sigma G_0 + G_0\Sigma G_0\Sigma G_0 + \cdots, \qquad \text{(Sup.2)}$$

where G_0 is the bare propagator, that is to say, the propagator in the medium without scatterer, and Σ is the self-energy describing the renormalization of single scattering center due to the interaction with the surrounding many-scatterers system.

Having introduced all necessary entities to the calculation of a diffuse intensity, let us now discuss

the analogy with DNA replication. As mentioned earlier, we are dealing with DNA replication process in a cell population. Eq. (Sup.1) now represents the total number $I(t)$ of fired origins at instant t in a cell and per genome length. As replication process is independent from one cell to an other, we assume that $|\psi(t)|^2 = \frac{1}{M}\delta(t)$, where M is the number of cells and $\delta(t)$ represents the fact that all cells in the volume are in the S phase. As there is not really an extra-cellular incoming signal that induces the replication process and that could be quantified, we set the scattering cross-section in Eq. (Sup.1) to $\sigma_{sc} = 1$. Under these assumptions, Eq. (Sup.1), reduces to

$$I(t) = \frac{n}{M}|G(t)|^2, \tag{Sup.3}$$

where $n = \frac{O_{total}}{L}$, O_{total} is the number of fired origins at the end of S phase (strong scatterers) and L is the length of the genome. The amplitude of the dressed Green's function $|G(t)|^2$ now represents the propagation of an origin from a not fired state (inactive state) to a fired state (active state). Therefore, the amplitude of the Green's function per cell ($\frac{|G(t)|^2}{M}$) is defined over the total number of accessible states (*i.e* $m_0 + O_{total}$). If there is no strong replication origins, in other words, if the number of accessible states in inactive state is equal to the number of states in active state ($O_{total} = m_0$), then the amplitude of the dressed Green's function that propagates an origin from an inactive to an active state is equal to 1. By analogy, this situation corresponds to a medium without scatterer, hence $G_0 = 1$. Therefore, following Dyson's Eq. (Sup.2), the amplitude of the dressed Green's function is $|G(t)|^2 = 1 + \Sigma + \Sigma^2 + \cdots$, where by analogy Σ represents the ability of a potential origin to fire. As previously discussed, the dressed Green's function is defined over the total number of states in the system. Along our definition of G_0, O_{total} corresponds to the number of strong firing replication origins and the other $(m_0 - O_{total})$ replication origins represent

the medium. Therefore, a potential origin is either a strong firing replication origin or a replication origin from the medium. As the firing ability ψ of a potential origin is the same for a strong firing origin and an origin from the medium, we express the self-energy as $\Sigma(t) = C\psi(t)$, where $C = \frac{2m_0}{m_0+O_{total}}$. Using Eqs. (Sup.2) and (Sup.3), after integration over the whole genome, we obtain the proportion $\rho(t) = \frac{O(t)}{m_0}$ of origin firing per cell at time t as:

$$\rho(t) = \frac{O_{total}}{2m_0} \sum_{\nu=0}^{O_{total}} (C\psi(t))^{\nu} - 1, \tag{Sup.4}$$

where the factor $\frac{1}{2}$ appears because in our analogy each firing event has been counted twice. In order to take into account all possible firing configurations, we expand the upper limit of the summation to infinity and approximate ρ to

$$\rho(t) \approx \frac{O_{total}}{2m_0}\left(\sum_{\nu=0}^{\infty} C^{\nu}\psi(t)^{\nu} - 1\right) \approx \frac{O_{total}}{2m_0}\left(\frac{1}{1-C\psi(t)} - 1\right). \tag{Sup.5}$$

2 Detailed derivation of Eq.(3)

To derive explicitly Eq. (3), we first differentiate Eq. (Sup.5):

$$\frac{d\rho(t)}{dt} = \frac{b^2}{2} C \frac{d\psi(t)}{dt} \frac{1}{(1-C\psi(t))^2}, \tag{Sup.6}$$

where $b = \sqrt{\frac{O_{total}}{m_0}}$. Then, remembering that $\psi(t) = \frac{O(t)}{m_0}$ represents the firing probability of an isolated origin, we use Eq. (1) to derive the following evolution equation:

$$\frac{d\psi(t)}{dt} = k'(t)\left(b^2 - \psi(t)\right)(1-\psi(t)), \tag{Sup.7}$$

where $k'(t) = m_0 k(t)$. To get a more compact form for the evolution equation of origin firing probability, we use the following change of variable $\phi(t) = 1 + \frac{2m_0}{O_{total}}\rho(t) = \frac{1}{1-\frac{\psi(t)}{a}}$, where $a = \frac{1}{C}$.

From Eqs. (Sup.6) and (Sup.7), we obtain :

$$\frac{d\phi}{dt} = \frac{k'(t)}{a}\left[a^2 - \left(a^2 - b^2\right)\phi^2(t)\right], \tag{Sup.8}$$

that corresponds to Eq. (3).

3 Is it necessary to consider the origin firing process as delocalised?

Historically the KJMA theory was developed by assuming that origins fire independently of each other, and that they only interact through traveling replication forks (passive replication)[7]. This theoretical framework was later modified to incorporate firing correlation among replication origins[8]; however, this *a posteriori* modification required an *a priori* knowledge of the nature and the range of correlations. As in this methodology the firing probability could be assigned to a single replication origin, it could be considered as a localized theory or a state theory (as was first mentioned by Kolmogrove himself[9]). The advantage of such a model is that after having defined in an explicit manner the correlation pattern among origins, one could extract from experimental data the local firing probability[10]. In our approach, Eq. (Sup.7) uses the same hypothesis as the original KJMA theory *i.e.* it represents the evolution equation for the firing probability in the case where the firing of a particular origin does not modify the firing probability of other origins. However, Eq. (Sup.8) represents the evolution equation for the collective firing of n replication origins at a time t and does not assume any hypothesis on their connectivity. Therefore, the firing probability $\rho(t)$ defined by Eq. (Sup.4) is distributed over n replication origins and in that sense it is delocalized. The advantage of such an approach is to allow us to extract the global temporal profile of probability of origin firing without any hypothesis on the interaction among replication origins. Let us note that, to recover the local probability $\psi(x,t)$ of origin firing, it would be necessary to assume a

particular configuration for the spatial distribution of replication origin firing.

Therefore, by representing the evolution of firing process as a bimolecular reaction (Eq. (1)), we have the possibility to verify mathematically if indeed the replication origins fire independently of each other or if their firing is correlated. One way to answer to this question is to verify which of the two evolution equations (Eqs. (Sup.7) and (Sup.8)) is valid under the experimental conditions. Using, as initial conditions, the fact that the probability of origin firing at the start of S phase is $\psi(t=0)=\rho(t=0)=0$, we solve analytically Eqs. (Sup.7) and (Sup.8) and find that:

$$\psi(t) = 2b^2 \frac{\tanh\left(\frac{(b^2-1)k_0}{2d_f\left(\frac{1}{d_w}+\frac{1}{2}\right)} t^{d_f\left(\frac{1}{d_w}+\frac{1}{2}\right)}\right)}{(b^2-1)+(b^2+1)\tanh\left(\frac{(b^2-1)k_0}{2d_f\left(\frac{1}{d_w}+\frac{1}{2}\right)} t^{d_f\left(\frac{1}{d_w}+\frac{1}{2}\right)}\right)}, \quad \text{(Sup.9)}$$

and

$$\rho(t) = 2b^4 \frac{\tanh\left(\frac{(1-b^2)k_0}{2d_f\left(\frac{1}{d_w}+\frac{1}{2}\right)} t^{d_f\left(\frac{1}{d_w}+\frac{1}{2}\right)}\right)}{1-b^4+(1-b^2)^2\tanh\left(\frac{(1-b^2)k_0}{2d_f\left(\frac{1}{d_w}+\frac{1}{2}\right)} t^{d_f\left(\frac{1}{d_w}+\frac{1}{2}\right)}\right)}. \quad \text{(Sup.10)}$$

Note that by using the expression of $\psi(t)$ (Eq. (Sup.9)) in Eq. (Sup.5), we obtain after some elementary algebra the same expression of $\rho(t)$ (Eq. (Sup.10)) as the one obtained by solving Eq. (Sup.8). As measured experimentally, at the end of S phase ($t = t_{end}$) all O_{total} origins have fired. Therefore, the probability of origin firing at the end of S phase is $\psi(t=t_{end}) = \rho(t=t_{end}) = \frac{O_{total}}{m_0} = b^2$. Thus at $t = t_{end}$, Eqs. (Sup.9) and (Sup.10) respectively reduce to:

$$\lim_{x\to -1} \tanh^{-1}(x) = -\infty = \frac{\left(b^2-1\right)k_0}{2d_f\left(\frac{1}{d_w}+\frac{1}{2}\right)} t_{end}^{d_f\left(\frac{1}{d_w}+\frac{1}{2}\right)}, \quad \text{(Sup.11)}$$

and

$$\tanh^{-1}\left(\frac{1-b^4}{2b^2-\left(1-b^2\right)^2}\right) = \frac{\left(1-b^2\right)k_0}{2d_f\left(\frac{1}{d_w}+\frac{1}{2}\right)} t_{end}^{d_f\left(\frac{1}{d_w}+\frac{1}{2}\right)}. \quad \text{(Sup.12)}$$

By remembering that t_{end}, d_f and d_w are measurable and therefore finite quantities, a close inspection of expressions (Sup.11) and (Sup.12) shows that while the value of k_0 is finite in Eq. (Sup.12),

this is not the case for Eq. (Sup.11) where k_0 should be equal to infinity, meaning that the firing of replication origins is highly efficient which is contradictory to experimental observations[11]. This is the demonstration that expression (Sup.9) for the probability of origin firing does not match with experimental observations, and more generally that the evolution Eq. (Sup.7) does not describe correctly the process of origin firing. Therefore, along the line defined in this work, to describe correctly the replication origin firing process one needs to delocalize this process over the whole potential replication origins distributed along the genome.

As the physical nature of a phenomenon is independent of the picture that is used to describe it, the results obtained here should also be valid for any picture that attempts to describe the replication process. Indeed, as discussed in previous sections, in the simplest form of KJMA model, an initiation event neither impedes nor favors origin initiation at another locus (localization of initiation). However, despite this hypothesis, Baker et al [10,12] have shown that the propagation of replication forks from fired replication origins creates an apparent correlation between firing time and efficiency of two distant fired replication origins due to passive replication. In that sense, in the KJMA model, the propagation of replication forks extends the effect of origin initiation at a particular locus to other distant loci thereby giving to this model a non-localized character. But, in contrast to our non-local modeling of DNA replication based on some analogy with scattering in inhomogeneous media, the KJMA model is based on a state theory where to understand the structure of local ($I\left(x,t\right)$) and/or whole genome averaged ($I\left(t\right)$) initiation function, it is necessary to assume a particular mechanism for the existence of correlations between the firing of replication origins[8].

Along the same lines of the approach we have used here to study the replication process, Gauthier and Bechhoefer [13] managed to reproduce the genome averaged rate of initiation $I(t)$ in early Xenopus embryos by assuming (i) that during the initial stage of S phase the firing process is reaction limited, while at the end of S phase it is diffusion limited and (ii) because of the fractal nature of the chromatin, the initiator factor undergoes a sub-diffusive dynamics. Indeed, the fact that the authors have assumed that the search process is influenced by the geometry of the chromatin amounts to introduce a memory in the dynamic of firing process. Thus the firing process at a particular locus will necessary depend on the other sites visited by a particular initiator factor consistent with a delocalized picture of firing process[14,15].

4 How sensitive is the model to the variation of parameter values?

To address this question we calculated Facs, $f_{DNA}(t)$), $I(t)$ and $N_f(t)$ profiles for both *S.cerevisiae* and human Hela cells by fixing all other variables to values used in the body of the article and only changing the value of one of the parameters (Figures S1-S10). While the all four profiles are sensitive to the values of the replication fork speed (v), the duration of S phase (t_{end}) and the parameters b, only $I(t)$ and $N_f(t)$ profiles are sensitive also to changes in the chromatin fractal dimension d_f and the dynamic dimension d_w.

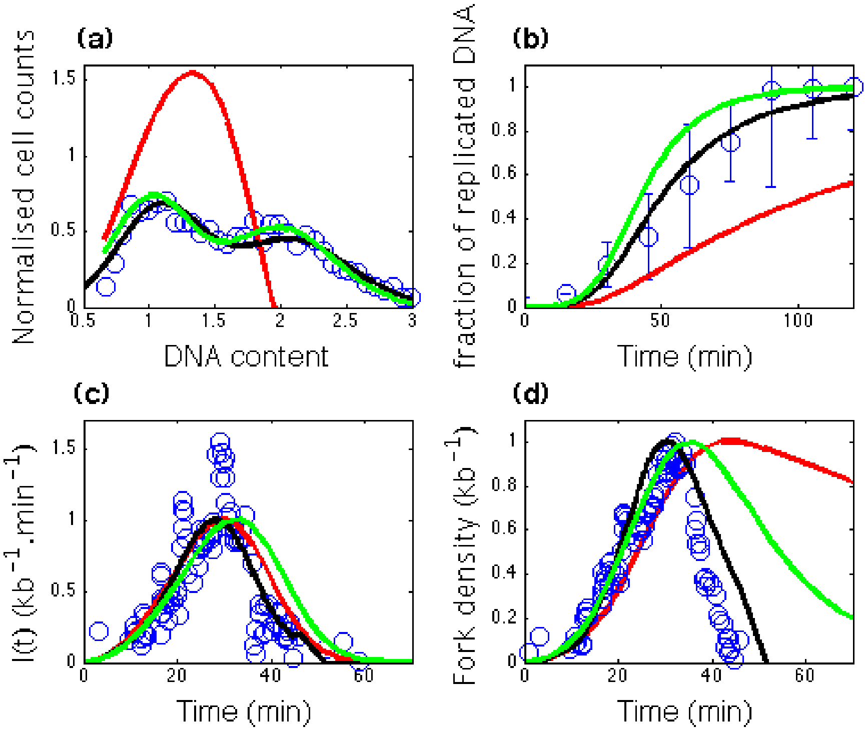

Figure S1: The open circles are experimental data and the solid lines are the calculated profiles. *S.cerevisiae* (data from Ma *et al.*[16]): (a) Facs profile, (b) $f_{DNA}\left(t\right)$), (c) $I\left(t\right)$ and (d) $N_f\left(t\right)$ for different values of the replication fork speed: $v = 0.5\ kb.min^{-1}$ (red), $1.68\ kb.min^{-1}$ (black) and $2.5\ kb.min^{-1}$ (green).

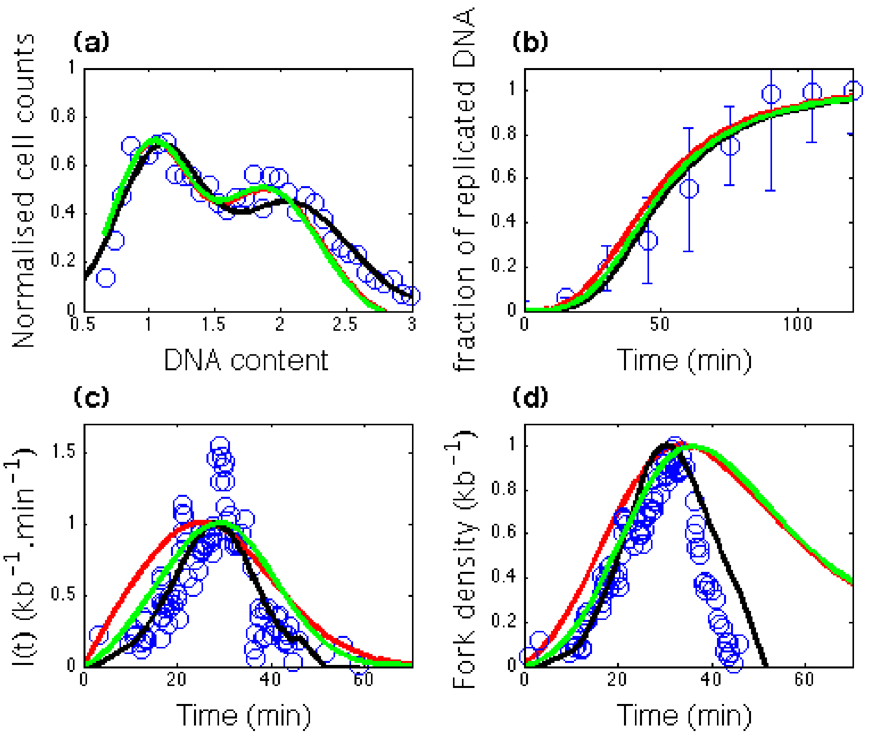

Figure S2: The open circles are experimental data and the solid lines are the calculated profiles. *S.cerevisiae* (data from Ma *et al.*[16]): (a) Facs profile, (b) $f_{DNA}(t)$), (c) $I(t)$ and (d) $N_f(t)$ for different values of the chromatin fractal dimension: $d_f = 2$ (red), 3 (black) and 2.5 (green).

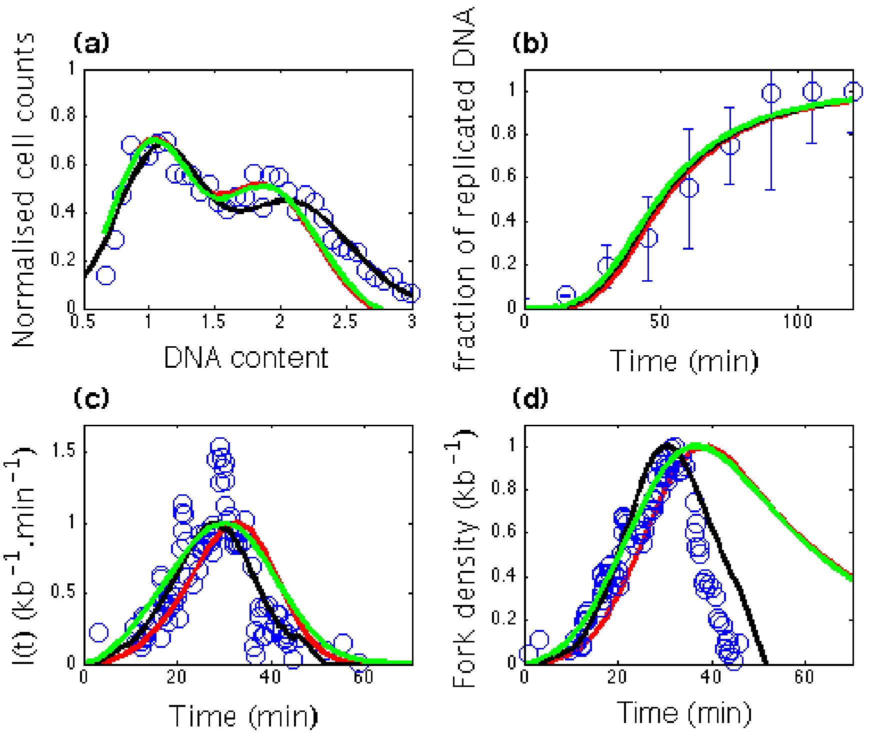

Figure S3: The open circles are experimental data and the solid lines are the calculated profiles. *S.cerevisiae* (data from Ma *et al.*[16]): (a) Facs profile, (b) $f_{DNA}(t)$), (c) $I(t)$ and (d) $N_f(t)$ for different values of the dynamics fractal dimension: $d_w = 1.5$ (red), 2 (black) and 2.5 (green).

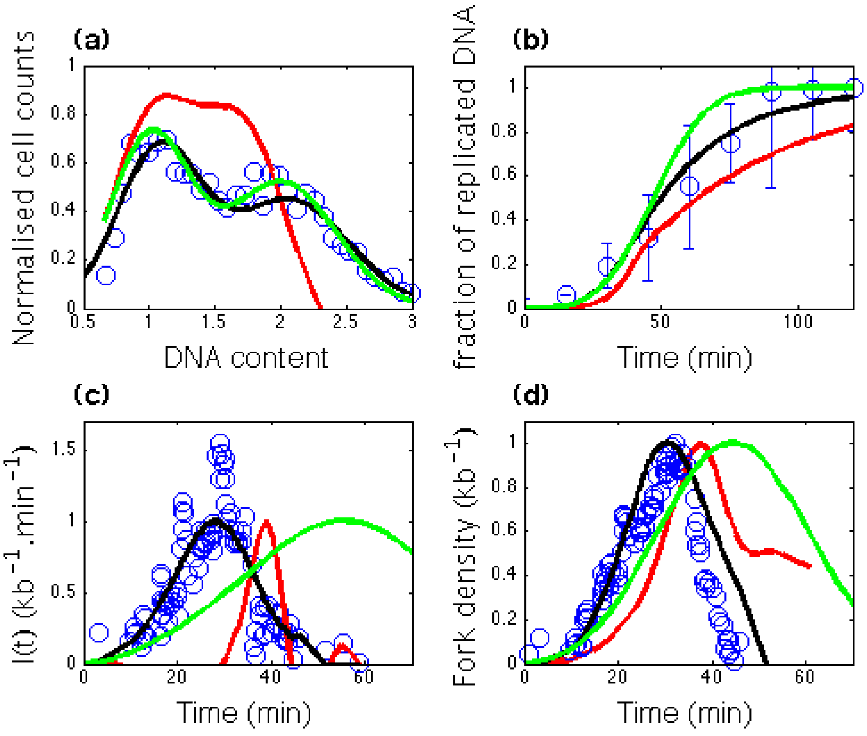

Figure S4: The open circles are experimental data and the solid lines are the calculated profiles. *S.cerevisiae* (data from Ma *et al.*[16]): (a) Facs profile, (b) $f_{DNA}(t)$), (c) $I(t)$ and (d) $N_f(t)$ for different values of $b^2 = \frac{O_{total}}{m_0}$: 0.43 (red), 0.52 (black) and 0.64 (green).

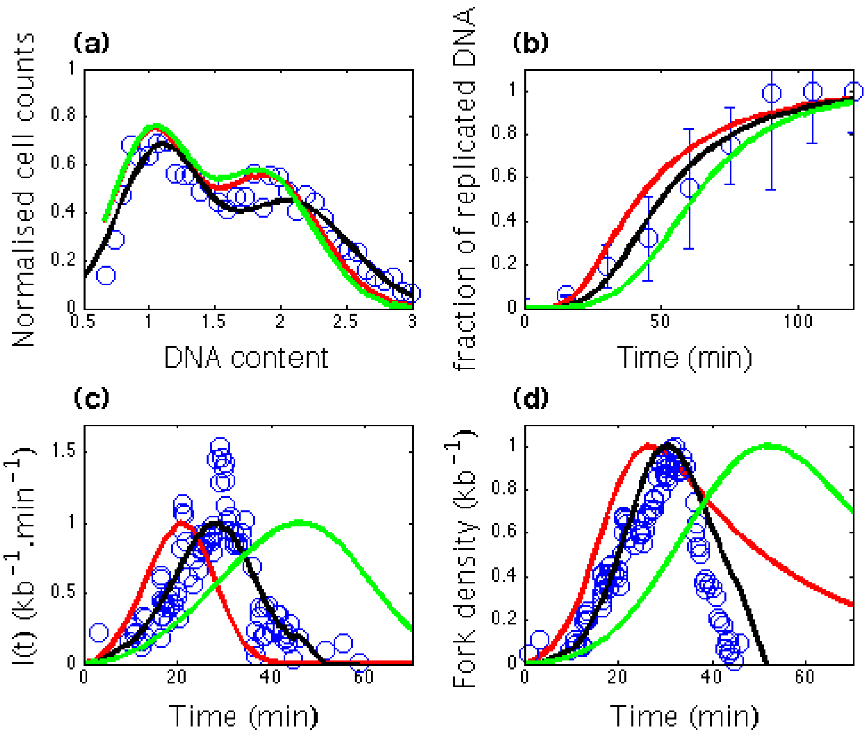

Figure S5: The open circles are experimental data and the solid lines are the calculated profiles. *S.cerevisiae* (data from Ma *et al.*[16]): (a) Facs profile, (b) $f_{DNA}(t)$), (c) $I(t)$ and (d) $N_f(t)$ for different values of S phase duration: $t_{end} = 29\ min$ (red), $42\ min$ (black) and $60\ min$ (green).

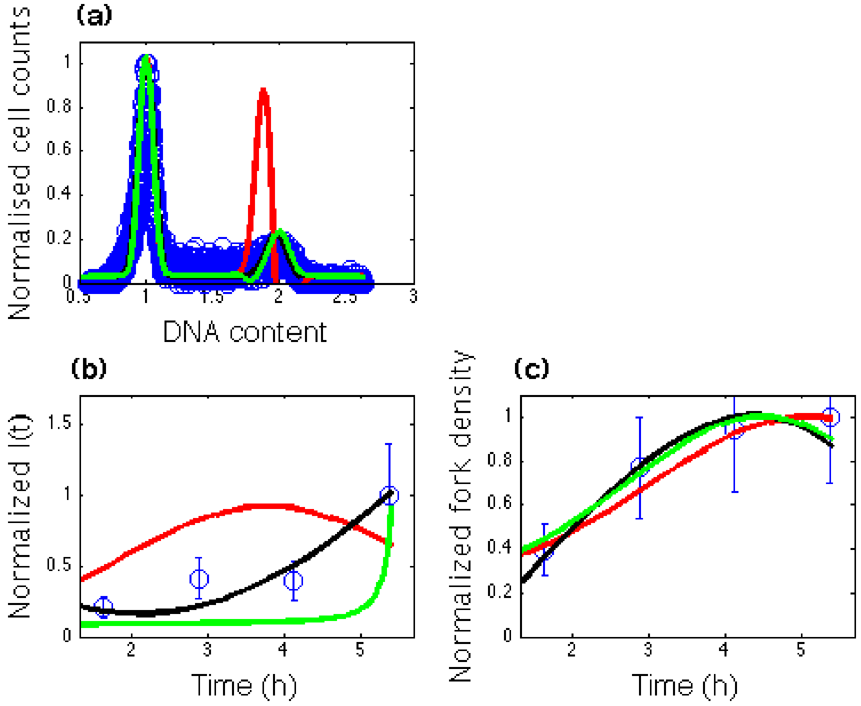

Figure S6: The open circles are experimental data and the solid lines are the calculated profiles. Hela (data from Guilbaud *et al.*[17]): (a) Facs profile, (b) $I(t)$ and (c) $N_f(t)$ for different values of the replication fork speed: $v = 0.8\ kb.min^{-1}$ (red), $1.1\ kb.min^{-1}$ (black) and $1.3\ kb.min^{-1}$ (green).

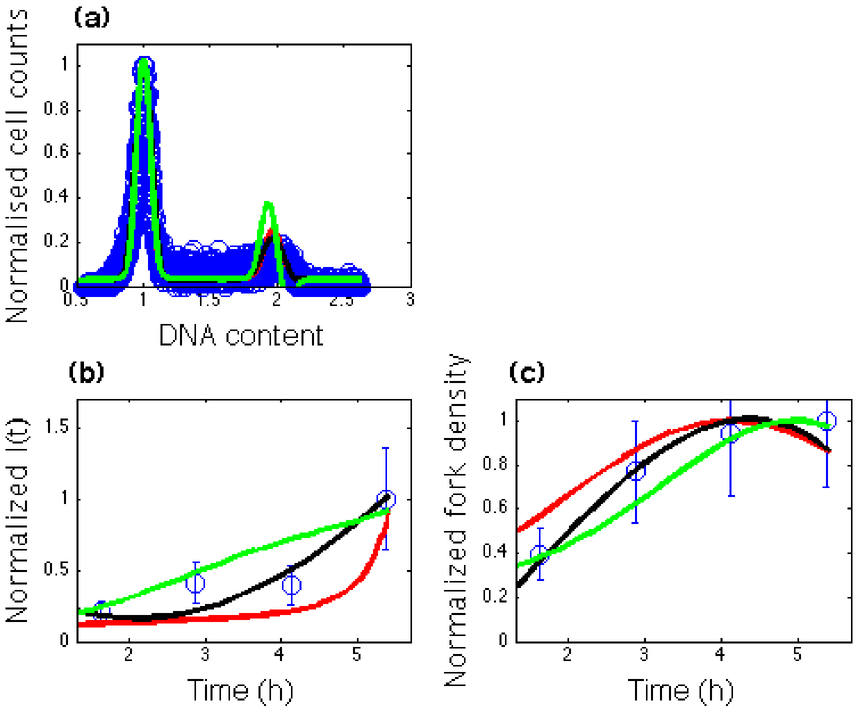

Figure S7: The open circles are experimental data and the solid lines are the calculated profiles. Hela (data from Guilbaud *et al.*[17]): (a) Facs profile, (b) $I(t)$ and (c) $N_f(t)$ for different values of the chromatin fractal dimension: $d_f = 2$ (red), 2.6 (black) and 3 (green).

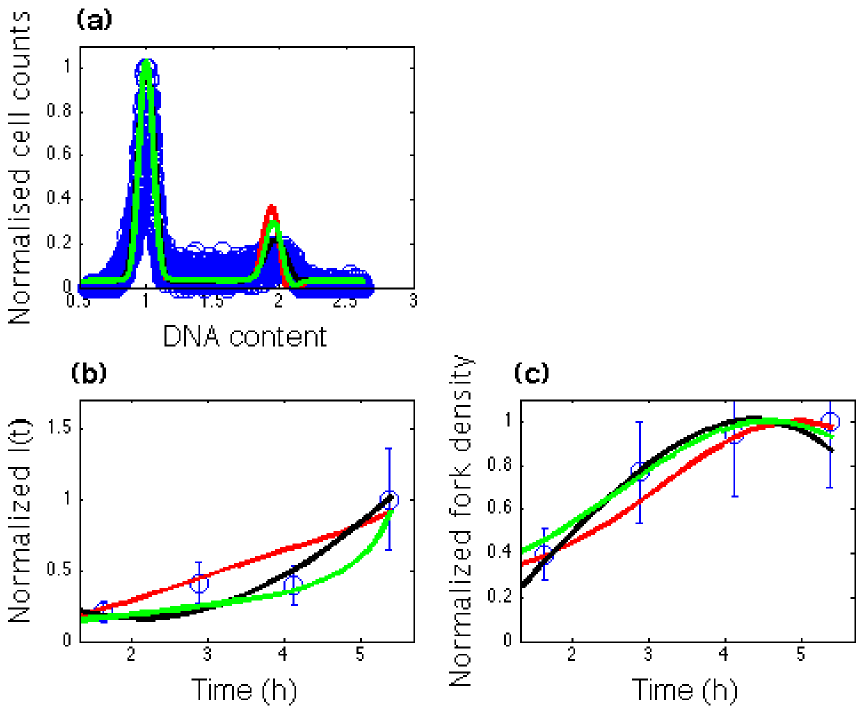

Figure S8: The open circles are experimental data and the solid lines are the calculated profiles. Hela (data from Guilbaud *et al.*[17]): (a) Facs profile, (b) $I(t)$ and (c) $N_f(t)$ for different values of the dynamics fractal dimension: $d_w = 2$ (red), 2.6 (black) and 3 (green).

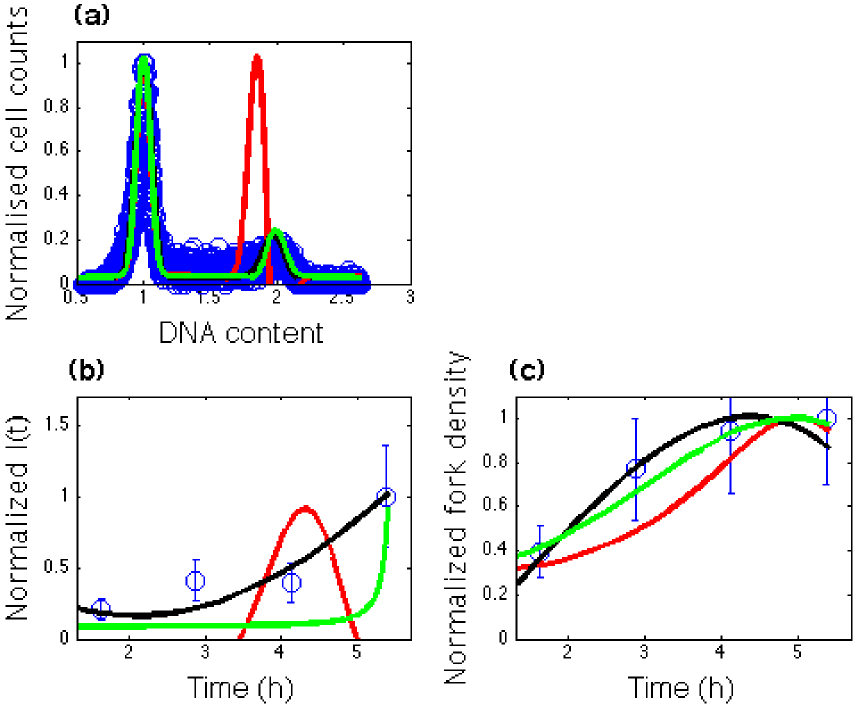

Figure S9: The open circles are experimental data and the solid lines are the calculated profiles. Hela (data from Guilbaud *et al.*[17]): (a) Facs profile, (b) $I(t)$ and (c) $N_f(t)$ for different values of $b^2 = \frac{O_{total}}{m_0}$: 0.42 (red), 0.54 (black) and 0.58 (green).

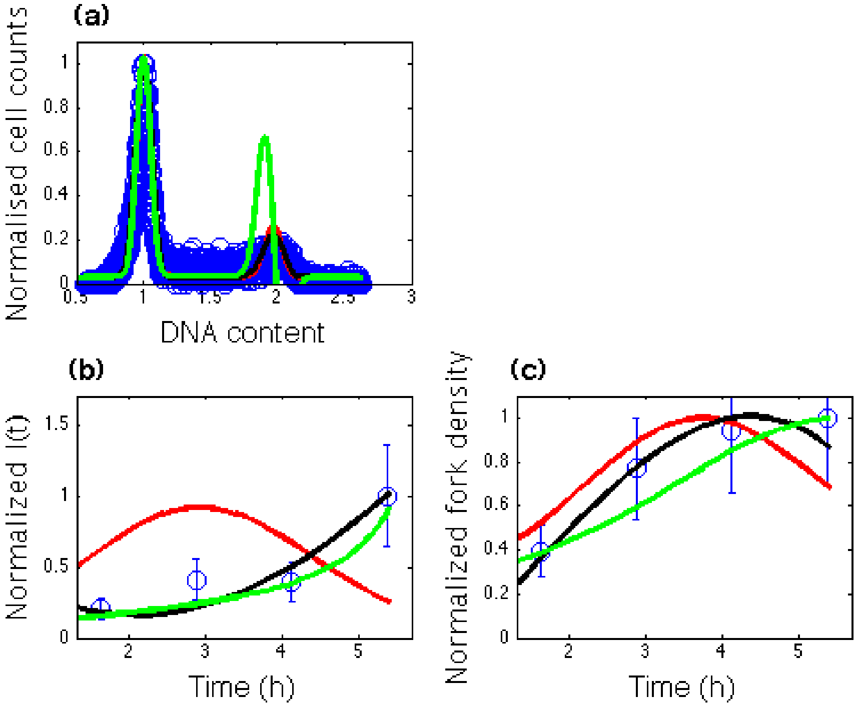

Figure S10: The open circles are experimental data and the solid lines are the calculated profiles. Hela (data from Guilbaud *et al.*[17]): (a) Facs profile, (b) $I(t)$ and (c) $N_f(t)$ for different values of S phase duration: $t_{end} = 360\ min$ (red), $480\ min$ (black) and $600\ min$ (green).

References:

1. van Rossum, M. C. W. & Nieuwenhuizen, T. M. Multiple scattering of classical waves: microscopy, mesoscopy, and diffusion. *Rev. Mod. Phys.* **71**, 313–371 (1999).

2. DePamphilis, M. L. *DNA Replication and Human Disease* (Cold Spring Harbor Laboratory Press, 2006).

3. Hyrien, O., Marheineke, K. & Goldar, A. Paradoxes of eukaryotic DNA replication: MCM proteins and the random completion problem. *Bioessays* **25**, 116–125 (2003).

4. Van Hove, L. Correlations in space and time and Born approximation scattering in systems of interacting particles. *Phys. Rev.* **95**, 249–262 (1954).

5. Mattuck, R. *A Guide to Feynman Diagrams in the Many-body Problem. Chapter X.* Dover Books on Physics Series (Dover Publications, Mineola 1976).

6. Fetter, A. & Walecka, J. *Quantum Theory of Many-particle Systems. Chapter XI and XII.* Dover Books on Physics (Dover Publications, Mineola 2003).

7. Herrick, J., Jun, S., Bechhoefer, J. & Bensimon, A. Kinetic model of DNA replication in eukaryotic organisms. *J. Mol. Biol.* **320**, 741–750 (2002).

8. Jun, S., Herrick, J., Bensimon, A. & Bechhoefer, J. Persistence length of chromatin determines origin spacing in xenopus early-embryo DNA replication: quantitative comparisons between theory and experiment. *Cell Cycle* **3**, 223–229 (2004).

9. Kolmogorov, A. N. On the statistical theory of the crystallization of metals. *Bull. Acad. Sci. USSR, Math. Ser* **1**, 355–359 (1937).

10. Baker, A., Audit, B., Yang, S. C.-H., Bechhoefer, J. & Arneodo, A. Inferring where and when replication initiates from genome-wide replication timing data. *Phys. Rev. Lett.* **108**, 268101 (2012).

11. Brewer, B. J. & Fangman, W. L. Mapping replication origins in yeast chromosomes. *Bioessays* **13**, 317–322 (1991).

12. Baker, A. & Bechhoefer, J. Inferring the spatiotemporal DNA replication program from noisy data. *Phys. Rev. E. Stat. Nonlin. Soft Matter Phys.* **89**, 032703 (2014).

13. Gauthier, M. G. & Bechhoefer, J. Control of DNA replication by anomalous reaction-diffusion kinetics. *Phys. Rev. Lett.* **102**, 158104 (2009).

14. Jeon, J.-H. & Metzler, R. Fractional brownian motion and motion governed by the fractional langevin equation in confined geometries. *Phys. Rev. E Stat. Nonlin. Soft Matter Phys.* **81**, 021103 (2010).

15. Kim, H.-J. Anomalous diffusion induced by enhancement of memory. *Phys. Rev. E. Stat. Nonlin. Soft Matter Phys.* **90**, 012103 (2014).

16. Ma, E., Hyrien, O. & Goldar, A. Do replication forks control late origin firing in saccharomyces cerevisiae? *Nucleic Acids Res.* **40**, 2010–2019 (2012).

17. Guilbaud, G. *et al.* Evidence for sequential and increasing activation of replication origins along replication timing gradients in the human genome. *PLoS Comput. Biol.* **7**, e1002322 (2011).
